# Is goal-directed fluid therapy based on dynamic variables alone sufficient to improve clinical outcomes among patients undergoing surgery? A meta-analysis

**DOI:** 10.1186/s13054-018-2251-2

**Published:** 2018-11-14

**Authors:** Qi-Wen Deng, Wen-Cheng Tan, Bing-Cheng Zhao, Shi-Hong Wen, Jian-Tong Shen, Miao Xu

**Affiliations:** 1 Department of Anesthesiology, the First Affiliated Hospital, Sun Yat-sen University, No.58, Zhongshan 2nd Road, Guangzhou, 510080 China; 2 Department of Endoscopy, Sun Yat-sen University Cancer Center, No. 651, Dongfeng East Road, Guangzhou, 510060 China; 3 Department of Anesthesiology, Nanfang Hospital, Southern Medical University, No. 1838, Guangzhou Avenue North, Guangzhou, 510515 China

**Keywords:** Goal-directed fluid therapy, Dynamic variables, Cardiac output, Surgery

## Abstract

**Background:**

Whether goal-directed fluid therapy based on dynamic predictors of fluid responsiveness (GDFTdyn) alone improves clinical outcomes in comparison with standard fluid therapy among patients undergoing surgery remains unclear.

**Methods:**

PubMed, EMBASE, the Cochrane Library and ClinicalTrials.gov were searched for relevant studies. Studies comparing the effects of GDFTdyn with that of standard fluid therapy on clinical outcomes among adult patients undergoing surgery were considered eligible. Two analyses were performed separately: GDFTdyn alone versus standard fluid therapy and GDFTdyn with other optimization goals versus standard fluid therapy. The primary outcomes were short-term mortality and overall morbidity, while the secondary outcomes were serum lactate concentration, organ-specific morbidity, and length of stay in the intensive care unit (ICU) and in hospital.

**Results:**

We included 37 studies with 2910 patients. Although GDFTdyn alone lowered serum lactate concentration (mean difference (MD) − 0.21 mmol/L, 95% confidence interval (CI) (− 0.39, − 0.03), *P* = 0.02), no significant difference was found between groups in short-term mortality (odds ratio (OR) 0.85, 95% CI (0.32, 2.24), *P* = 0.74), overall morbidity (OR 1.03, 95% CI (0.31, 3.37), *P* = 0.97), organ-specific morbidity, or length of stay in the ICU and in hospital. Analysis of trials involving the combination of GDFTdyn and other optimization goals (mainly cardiac output (CO) or cardiac index (CIx)) showed a significant reduction in short-term mortality (OR 0.45, 95% CI (0.24, 0.85), *P* = 0.01), overall morbidity (OR 0.41, 95% CI (0.28, 0.58), *P* < 0.00001), serum lactate concentration (MD − 0.60 mmol/L, 95% CI (− 1.04, − 0.15), *P* = 0.009), cardiopulmonary complications (cardiac arrhythmia (OR 0.58, 95% CI (0.37, 0.92), *P* = 0.02), myocardial infarction (OR 0.35, 95% CI (0.16, 0.76), *P* = 0.008), heart failure/cardiovascular dysfunction (OR 0.31, 95% CI (0.14, 0.67), *P* = 0.003), acute lung injury/acute respiratory distress syndrome (OR 0.13, 95% CI (0.02, 0.74), *P* = 0.02), pneumonia (OR 0.4, 95% CI (0.24, 0.65), *P* = 0.0002)), length of stay in the ICU (MD − 0.77 days, 95% CI (− 1.07, − 0.46), *P* < 0.00001) and in hospital (MD − 1.18 days, 95% CI (− 1.90, − 0.46), *P* = 0.001).

**Conclusions:**

It was not the optimization of fluid responsiveness by GDFTdyn alone but rather the optimization of tissue and organ perfusion by GDFTdyn and other optimization goals that benefited patients undergoing surgery. Patients managed with the combination of GDFTdyn and CO/CI goals might derive most benefit.

**Electronic supplementary material:**

The online version of this article (10.1186/s13054-018-2251-2) contains supplementary material, which is available to authorized users.

## Background

Inappropriate fluid administration in the intraoperative period is associated with a risk of hypovolemia or overload. It then causes tissue hypoxia and postoperative organ dysfunction. The postoperative complications have a huge impact on short-term and long-term mortality. The occurrence of these complications could reduce median survival by 69% [1]. Moreover, the increased morbidity and mortality is associated with a high healthcare cost [2]. Correcting tissue hypoxia is a crucial step to improve the prognosis of patients undergoing surgery.

Occult tissue hypoxia still occurs despite the normalization of standard physiologic variables, such as heart rate, blood pressure, central venous pressure (CVP) and urine output [3, 4]. Goal-directed fluid therapy based on dynamic variables (GDFTdyn) is defined as a spectrum of fluid management strategies reaching optimal preload by monitoring variables derived from cardiorespiratory interaction. These variables include stroke volume variation (SVV), systolic pressure variation (SPV), pulse pressure variation (PPV) and pleth variability index (PVI). They have emerged to target tissue perfusion in recent years. They are believed to be the markers of positions on the Frank-Starling curve, which are proportional to the degree of preload dependency. Compared with stroke volume optimization requiring quantification of the percentage change in stroke volume or oxygen delivery optimization requiring frequent calculations of oxygen delivery related variables, GDFTdyn is perceived to be more direct and less time-consuming. It is thought to be more convenient for healthcare providers to know whether a patient is a fluid responder or not. Moreover, as arterial cannulation and pulse oximeter are routinely used in moderate to high-risk patients undergoing surgery, these dynamic variables are easy to obtain and well-tolerated by patients. These advantages of GDFTdyn make it possible to be widely used in clinical practice.

Numerous clinical trials and systematic reviews have evaluated the efficacy and safety of GDFTdyn in patients undergoing surgery [5–9]. However, most of these clinical trials are of small sample size and the results of them contradict each other. On the other hand, there may be significant heterogeneity and methodological flaws in the previous meta-analyses. Especially, existing meta-analyses have failed to account for nonuniform application of other combined optimization goals in the GDFTdyn arms. These combined optimization goals are variables not derived from cardiorespiratory interaction, such as variables of flow, cardiac output (CO) or cardiac index (CIx). They might serve to confound the final results. As a result, whether GDFTdyn alone improves clinical outcomes among patients undergoing surgery or not remains uncertain.

Therefore, we performed the meta-analysis to determine the effects of GDFTdyn in comparison with standard fluid therapy on clinical outcomes among adult patients undergoing surgery. Especially, we compared GDFTdyn alone and GDFTdyn with other optimization goals separately to better address the question.

## Methods

The meta-analysis was conducted following the recommendations of Cochrane Handbook for Systematic Reviews of Interventions [10], and reported following Preferred Reporting Items for Systematic Reviews and Meta-Analyses (PRISMA) guidelines [11] (see Additional file 1). The protocol of the study has been registered in PROSPERO (CRD42018106439).

### Literature search

A systematic search of PubMed, EMBASE, the Cochrane Library and ClinicalTrials.gov was performed independently by two authors (QWD and WCT) to identify relevant studies in any language published from inception to 1 September 2018. Electronic search keywords were goal directed (goal targeted, goal oriented), and fluid management (fluid optimization, fluid therapy), surgery (operation, intraoperative, perioperative). Additional studies were identified by reviewing the reference lists of previous systematic reviews. The search strategy used in PubMed was as follow: (1) “goal directed”; (2) “goal targeted”; (3) “goal oriented”; (4) 1 or 2 or 3; (5) fluid; (6) hemodynamic; (7) haemodynamic; (8) 5 or 6 or 7; (9) management; (10) optimization; (11) therapy; (12) 9 or 10 or 11; (13) 8 and 12; (14) surg*; (15) operat*; (16) intraoperative*; (17) perioperative*; (18) 14 or 15 or 16 or 17; (19) 4 and 13 and 18.

### Study selection

After excluding studies based on title and abstract screening, two authors (QWD and BCZ) independently reviewed the full texts of the remaining studies. Consensus was resolved by the third author (WCT) when disagreement occurred. Studies were considered eligible if they met the following inclusion criteria.

### Type of participants

Adult patients (> 18 years old) undergoing surgery were included as participants. The patients were defined as high risk when they fulfilled at least one of the patient-related or surgery-related criteria. The patient-related criteria were age >60 years or American Society of Anesthesiologists (ASA) score ≥ 3 due to any reason. The surgery-related criteria were high-risk surgeries defined by original studies and by European Society of Cardiology/European Society of Anesthesiology (ESC/ESA) guidelines [12], including emergency surgery, cardiac surgery, major vascular surgery, major abdominal surgery, or surgeries with presumed blood loss >20% of blood volume.

### Type of intervention

The intervention was defined as GDFT based on dynamic variables derived from cardiorespiratory interaction, including SVV, SPV, PPV and PVI. Variables not derived from cardiorespiratory interaction were considered as other optimization goals, such as CO, CI, and oxygen delivery.

### Type of comparison

Comparison of the effects of GDFTdyn with those of standard fluid management was considered. Standard fluid management was defined as fluid management based on standard physiologic variables, such as heart rate, blood pressure, central venous pressure (CVP) or urine output.

### Type of outcome measures

The primary outcomes were short-term mortality and overall morbidity. Short-term mortality was defined as 30-day or hospital mortality. Overall morbidity was defined as the proportion of patients with one or more postoperative complications. The secondary outcomes were serum lactate concentration at the end of surgery, organ-specific morbidity (neurological, cardiovascular, pulmonary, abdominal and renal complications), and length of stay in the ICU and in hospital. The organ-specific morbidity was defined as the proportion of patients with an organ-specific complication. These complications included neurological (stroke), cardiovascular (arrhythmia, myocardial infarction, heart failure/cardiovascular dysfunction), pulmonary (acute lung injury/acute respiratory distress syndrome (ALI/ARDS), pneumonia, pulmonary embolism), abdominal (gastrointestinal (GIT) bleeding, GIT obstruction) and renal (acute kidney injury (AKI), renal failure with dialysis) complications.

Studies were excluded if they did not report any of these clinical outcomes.

### Data extraction

Data were independently extracted to a predesigned form by two authors (SHW and JTS). The following variables were collected: first author, year of publication, study design, patient demographics (age, sample size, ASA class, high or moderate risk), surgical variables (surgical procedure, duration of surgery, estimated blood loss), intraoperative fluid administration (GDFTdyn, other optimization goals, monitoring devices, fluid management), and outcomes (short-term mortality, overall morbidity, serum lactate concentration at the end of surgery, postoperative organ-specific complications, length of stay in ICU and hospital).

### Quality assessment

The Cochrane Collaboration’s tool for assessing risk of bias was applied. It focuses upon selection bias, performance bias, detection bias, attrition bias, and reporting bias.

### Statistical analysis

We performed two separate analyses by pooling data from RCTs comparing GDFTdyn alone or GDFTdyn with other optimization goals with standard fluid therapy (analysis 1: GDFTdyn alone versus standard fluid therapy; analysis 2: GDFTdyn with other optimization goals versus standard fluid therapy, respectively). We divided the included studies into these two groups according to the combination of other optimization goals. Note that we did not take heart rate, blood pressure, CVP, and urine output into consideration of other optimization goals because normalization of them could not prevent the occurrence of occult tissue hypoxia [3, 4]. Sensitivity analysis was conducted after excluding studies with high risk of bias. Subgroup analyses were conducted according to the type of surgery (cardiac or non-cardiac), patient risk (high or moderate risk), fluid management (fluid with or without inotropes), and monitoring devices (minimally invasive or non-invasive).

Statistical analysis was performed using Review Manager 5.3 software (Cochrane Collaboration, Denmark). Dichotomous data outcomes were analyzed using Mantel-Haenszel random-effects model and results presented as odds ratios (OR) with 95% confidence intervals (CI). Continuous data outcomes were analyzed using inverse variance random-effects modeling and quoted as mean differences (MD) with 95% CIs. A statistically significant difference between groups was considered to be present if the pooled 95% CI did not include 0 for respective MD or 1 for respective OR. Statistical heterogeneity was assessed by *I*-square test and considered to be significant if *I*-square was > 75%.

## Results

### Study selection and characteristics

After removal of duplicates, a total of 794 studies remained: 81 studies were reviewed in full and 37 studies finally met the inclusion criteria. The process of literature searching, screening and selection is presented in Additional file 2. The 37 studies included a total of 2910 patients, 1456 in the GDFTdyn arm and 1454 in the standard fluid therapy arm [13–49]. Patients in 27 studies were defined as high risk due to patient-related or surgery-related reasons. Of all included studies, 20 studies were based in abdominal surgery, 5 in cardiovascular, 3 in neurological, 2 in head and neck, 2 in thoracic, 1 in orthopedic and 1 in urologic surgery. Analysis 1 included 11 studies and analysis 2 included 26 studies. SVV, PVV, SPV and PVI were measured as GDFTdyn endpoints. CO or CI was the common or even the only goal except for GDFTdyn endpoints in almost all studies included in analysis 2. The characteristics of the included studies are summarized in Table 1.

**Table 1:** Main characteristics of included studies

Study	Type of surgery	Patients (GDFT), *n*	Patients (control), *n*	Risk	Age, years	GDFTdyn goals	Other goals	Monitoring devices	Interventions
Benes J 2010 [13]	Major abdominal	60	60	High	> 18	SVV < 10%	CI 2.5–4 L/min/m^2^	FloTrac/Vigileo	Fluid inotropes vasopressors
Broch O 2016 [14]	Major abdominal	39	40	High	> 18	PPV < 10%^a^	CI > 2.5 L/min/m^2^	Nexfin^b^	Fluid inotropes vasopressors
Buettner M 2008 [15]	Major abdominal	40	40	High	> 18	SPV < 10%	–	PiCCOplus	Fluid vasopressor
Cesur S 2018 [16]	Abdominal	35	35	Moderate	> 18	PVI < 13%	–	Masimo Radical 7^b^	Fluid vasopressors
Colantonio L 2015 [17]	Major abdominal	38	42	High	> 18	SVV < 15%	SVI > 35 mL/min/m^2^ CI > 2.5 L/min/m^2^	FloTrac/Vigileo	Fluid inotropes
Correa-Gallego C 2015 [18]	Major abdominal	69	66	High	NR	SVV < 15%	CO > 4 L/min CI > 2 L/min/m^2^	FloTrac	Fluid
Demirel İ 2018 [19]	Abdominal	30	30	Moderate	> 18	PVI < 14%	–	Masimo Co.^b^	Fluid vasopressors
Elgendy MA 2017 [20]	High risk	43	43	High	NR	SVV < 12%	CI > 2.5 L/min/m2	FloTrac/Vigileo	Fluid inotropes vasopressors
Fellahi JL 2015 [21]	Cardiac	48	44	High	> 18	SVV ≤11%	CI > 2.4 L/min/m^2^	Endotracheal cardiac output monitor	Fluid inotropes
Forget P 2010 [22]	Abdominal	41	41	Moderate	> 18	PVI < 13%	–	Masimo Co.^b^	Fluid vasopressors
Funk DJ 2015 [23]	Major vascular	20	20	High	> 18	SVV < 13%	CI > 2.2 L/min/m^2^	FloTrac/Vigileo	Fluid inotropes vasopressors
Goepfert MS 2013 [24]	Cardiac	50	50	High	> 18	SVV < 10%	CI > 2 L/min/m^2^	PiCCOplus	Fluid inotropes vasopressors
Hand WR 2016 [25]	Head and neck	47	47	Moderate	NR	SVV < 13%	CI > 3 L/min/m^2^ SVR > 800 dynes/s/cm^5^/m^2^	FloTrac/Vigileo	Fluid inotropes vasopressors
Harten J 2008 [26]	Emergency abdominal	14	15	High	> 50	PPV < 10%	–	Lidco plus	Fluid
Kapoor PM 2008 [27]	Cardiac	13	14	High	NR	SVV < 10%^a^	CI 2.5–4.2 L/min/m^2^ SVI 30-65 mL/beat/m^2^ SVRI: 1500–2500 dynes/s/cm^5^/m^2^ DO_2_ 450–600 mL/min/m^2^ ScVO_2_ > 70%	FloTrac/Vigileo	Fluid inotropes vasoactives
Kapoor PM 2016 [28]	Cardiac	60	60	High	NR	SVV < 10%^a^	CI 2.5–4.2 L/min/m^2^ SVI 30–65 mL/beat/m^2^ SVRI 1500–2500 dynes/s/cm^5^/m^2^ DO_2_ 450–600 mL/min/m^2^ ScVO_2_ > 70% Hct > 30% ScVO_2_ > 70%	FloTrac/Vigileo	Fluid inotropes vasodilators
Kim HJ 2018 [29]	Head and neck	31	31	Moderate	20–80	SVV < 12%	CI > 2.5 L/min/m^2^	FloTrac/Vigileo	Fluid inotropes vasodilators
Kumar L 2016 [30]	Major abdominal	30	30	High	> 18	SVV < 10%	CI ≥2.5 L/min/m^2^ O_2_ER ≤ 27%	FloTrac/Vigileo	Fluid inotropes vasopressors
Lai CW 2015 [31]	Major abdominal	109	111	High	NR	SVV < 10%	–	LiDCOrapid	Fluid
Liang M 2017 [32]	Urologic	30	30	High	60–80	SVV 8%–13%	DO_2_I ≥ 500 mL/min/m^2^	FloTrac/Vigileo	Fluid inotropes
Lopes MR 2007 [33]	High risk	17	16	High	> 18	PPV < 10%	–	IBPplus	Fluid
Luo J 2017 [34]	Craniotomy	73	72	High	> 18	SVV < 15%	CI > 2.5 L/min/m^2^	FloTrac/Vigileo	Fluid inotropes vasopressors
Mayer J 2010 [35]	Major abdominal	30	30	High	> 18	SVV < 12%	CI > 2.5 L/min/m^2^ SVI > 35 mL/m^2^	FloTrac/Vigileo	Fluid inotropes vasopressors
Peng K 2014 [36]	Major orthopedic	40	40	High	> 18	SVV < 10%/14%	–	FloTrac/Vigileo	Fluid vasopressors
Pösö T 2014 [37]	Abdominal	30	20	Moderate	NR	SVV < 12%	CI ≥2.0 L/min/m^2^	FloTrac/Vigileo	Fluid inotropes vasopressors
Ramsingh DS 2013 [38]	Major abdominal	18	20	High	> 18	SVV < 12%	–	FloTrac/Vigileo	Fluid
Salzwedel C 2013 [39]	Major abdominal	79	81	High	NR	PPV < 10%	CI > 2.5 L/min/m^2^	ProAQT	Fluid inotropes vasopressors
Scheeren TW 2013 [40]	High risk	26	26	High	> 18	SVV < 10%	SV rise > 10%	FloTrac/Vigileo	Fluid
Stens J 2015 [41]	Abdominal	13	18	Moderate	> 18	PPV < 12%	CI > 2.5 L/min/m^2^	Nexfin^b^	Fluid inotropes vasopressors
Sundaram SC 2016 [42]	Intracranial tumor	30	30	High	20–80	PPV < 13%	–	Phillips Intellivue MP50	Fluid vasopressors
Weinberg L 2017 [43]	Major abdominal	26	26	High	> 18	SVV < 20%	CI > 2.0 L/min/m^2^ PaO_2_ > 100 mmHg Hb > 8 g/dL *T* > 36 °C	FloTrac/Vigileo	Fluid inotropes vasopressors
Wu J 2017 [44]	Intracranial tumor	33	30	High	NR	SVV < 12%	CI > 2.5 L/min/m^2^	FloTrac/Vigileo	Fluid inotropes vasopressors
Xu H 2017 [45]	Thoracic	84	84	Moderate	18–60	SVV 10– 13%	CI > 2.5 L/min/m^2^	FloTrac/Vigileo	Fluid inotropes vasopressors
Yu Y 2014 [46]	Abdominal	15	15	Moderate	20–65	PVI < 13%	–	Masimo Radical 7^b^	Fluid vasopressors
Zhang J 2013 [47]	Thoracic	30	30	Moderate	18–60	SVV 9– 11%	CI > 2.5 L/min/m^2^	FloTrac/Vigileo	Fluid inotropes
Zheng H 2013 [48]	Major abdominal	30	30	High	60–80	SVV < 12%	CI > 2.5 L/min/m^2^ SVI > 35 mL/m^2^	FloTrac/Vigileo	Fluid inotropes vasopressors
Zheng LS 2016 [49]	Major abdominal	39	37	High	65–90	SVV < 12%	CI > 2.5 L/min/m^2^	FloTrac/Vigileo	Fluid vasopressors

*CIx* cardiac index, *CO* cardiac output, *DO*_*2*_ oxygen delivery, *GDFT* goal-directed fluid therapy, *GDFTdyn* goal-directed fluid therapy based on dynamic variables, *Hb* hemoglobin, *Hct* Red blood cell specific volume, *NR* not reported, *O*_*2*_*ER* O_2_ extraction rate, *PaO*_*2*_ partial pressure of oxygen, *PPV* pulse pressure variation, *PVI* pleth variability index; *ScVO*_*2*_ systemic central venous oxygen saturation, *SPV* systolic pressure variation, *SV* stroke volume, *SVI* stroke volume index, *SVR* systemic vascular resistance, *SVRI* systemic vascular resistance index, *SVV* stroke volume variation, *T* temperature

^a^Algorithms for GDFTdyn in these studies were performed intraoperatively and shortly after surgery, while others were performed only intraoperatively

^b^Monitoring devices in these studies were non-invasive, while others were minimally invasive

### Quality assessment

Risk of bias was assessed by the Cochrane Collaboration’s tool. The methodological quality of the included studies is summarized in Additional file 3. Random sequence generation was clearly reported in 30 of the included studies and allocation concealment in 22 studies: 17 of the studies clearly stated the blinding of participants, and 24 of the studies clearly reported blinding of the outcome assessment. Incomplete outcome data were not clearly reported in six studies. Selective reporting was found only in one study.

### Meta-analyses

#### Analysis 1: GDFTdyn alone versus standard fluid therapy

##### Primary outcomes

**Fig. 1:**
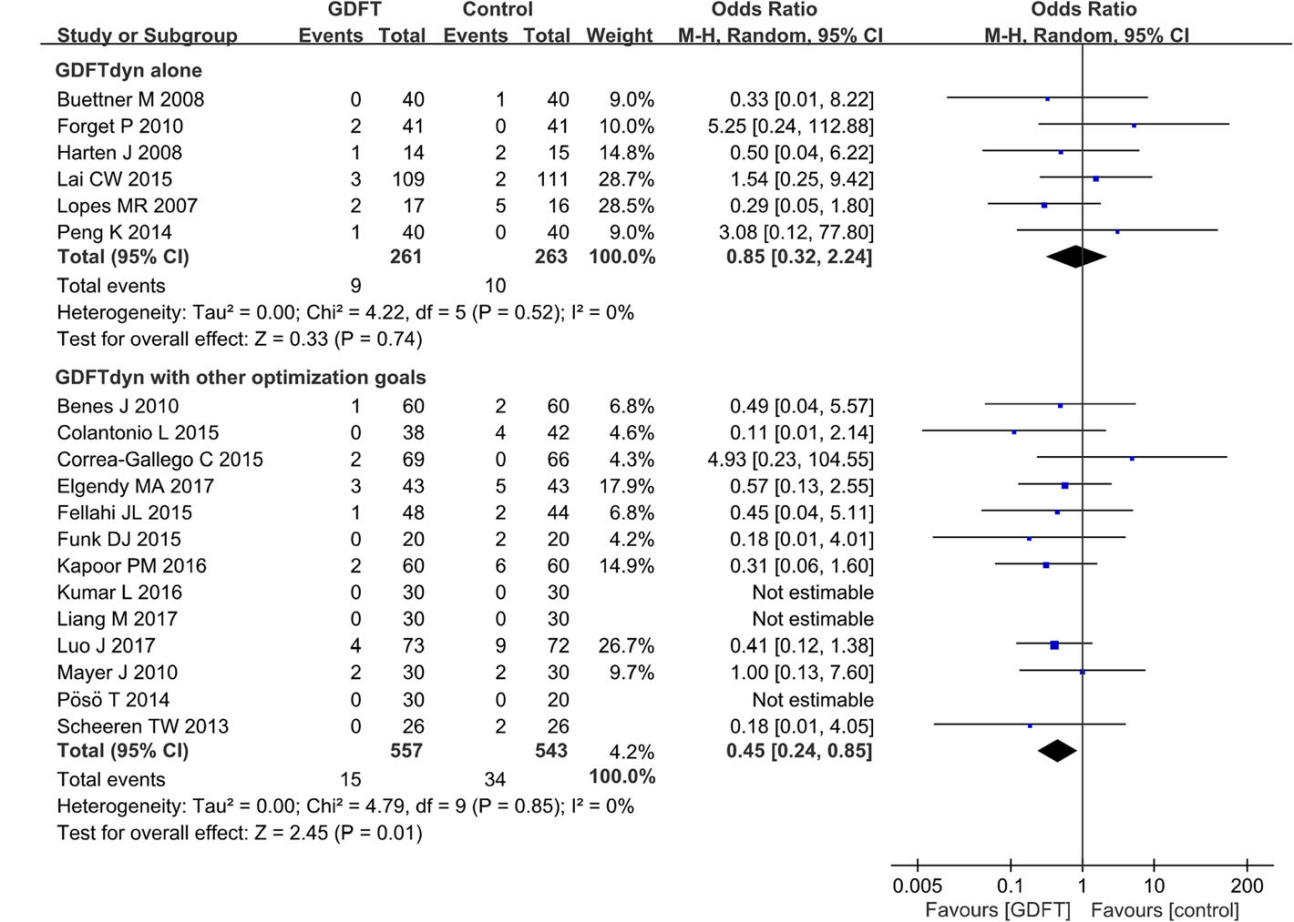
Short-term mortality. GDFT, goal-directed fluid therapy; M-H, Mantel-Haenszel

Six studies including 524 patients reported postoperative short-term mortality. The meta-analysis of these trials showed no significant difference between the patients managed with GDFTdyn alone and those with standard fluid therapy (OR 0.85, 95% CI (0.32, 2.24), *P* = 0.74, I^2^ = 0%) (Fig. 1). Sensitive analysis excluding studies with high risk of bias also showed no significant difference between two groups (Additional file 4). No significant difference was found between two groups among any subgroup analyses (Table 3).

**Fig. 2:**
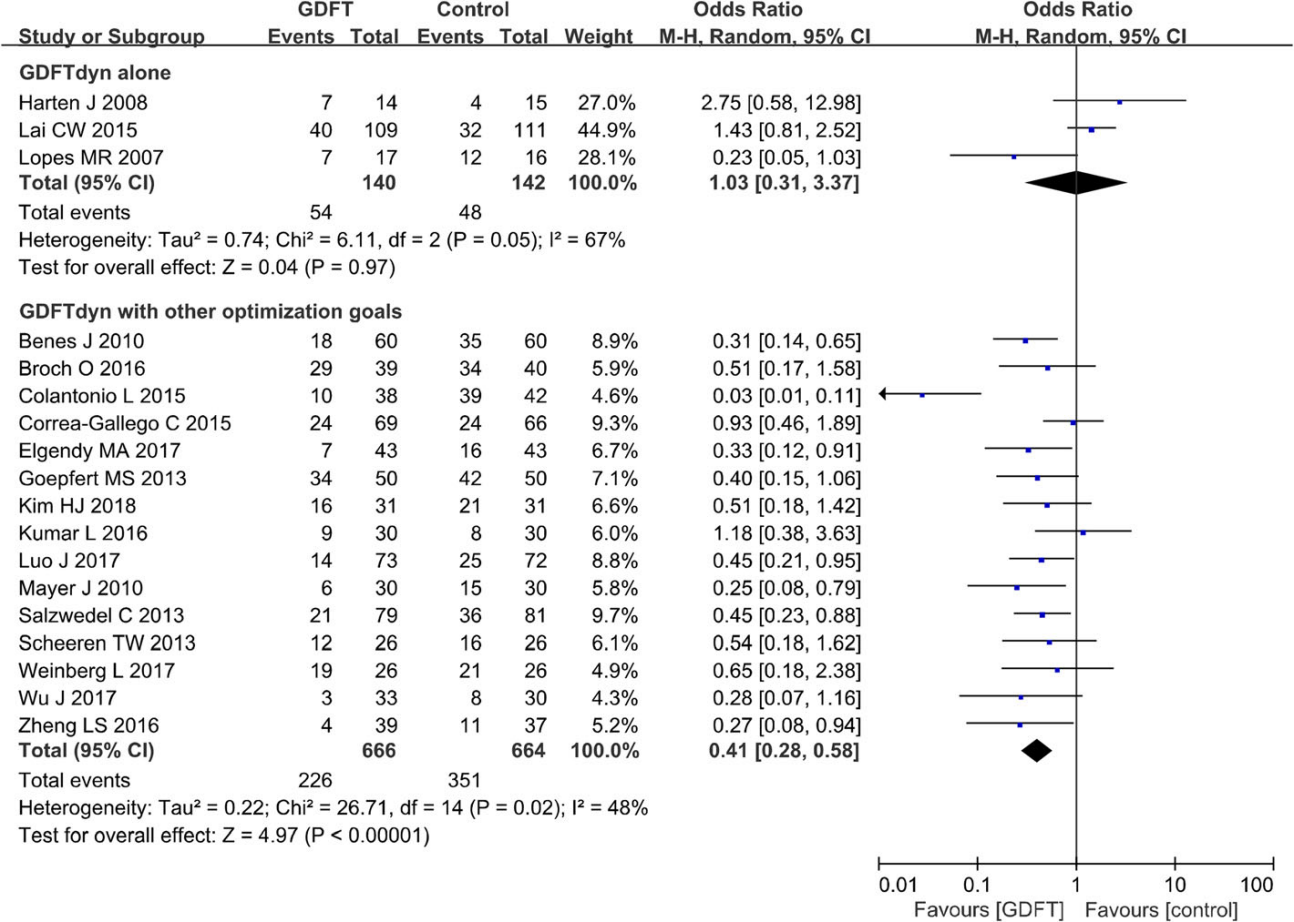
Overall morbidity. GDFT, goal-directed fluid therapy; M-H, Mantel-Haenszel

Three studies including 282 patients reported postoperative overall morbidity. No significant difference was observed between GDFTdyn alone and standard fluid therapy group (OR 1.03, 95% CI (0.31, 3.37), *P* = 0.97, I^2^ = 67%) (Fig. 2). Sensitive analysis excluding studies with high risk of bias also showed no significant difference between two groups (Additional file 5). No significant difference was found between two groups in any subgroup analyses (Table 3).

### Secondary outcomes

**Fig. 3:**
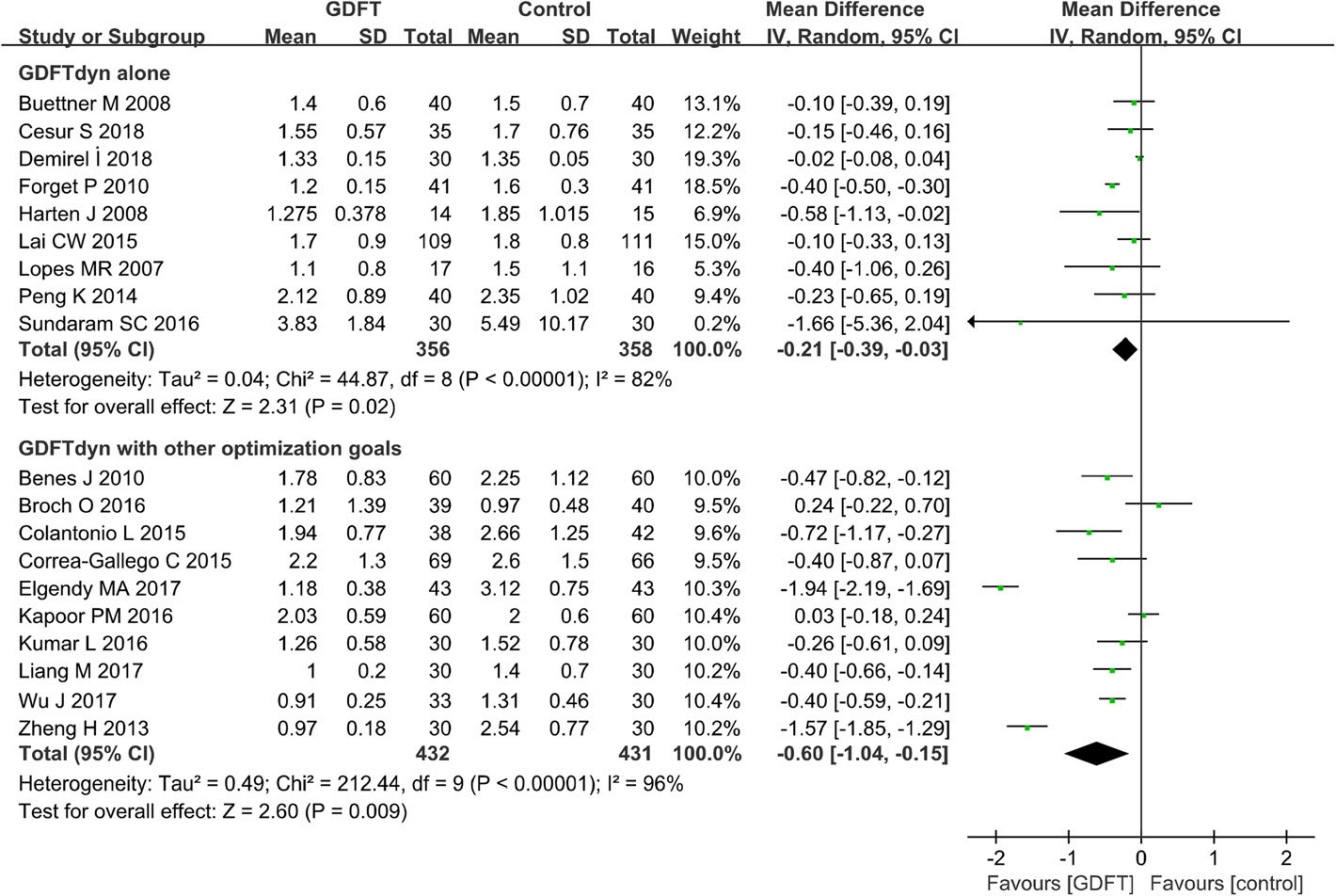
Serum lactate at the end of surgery. GDFT, goal-directed fluid therapy

**Fig. 4:**
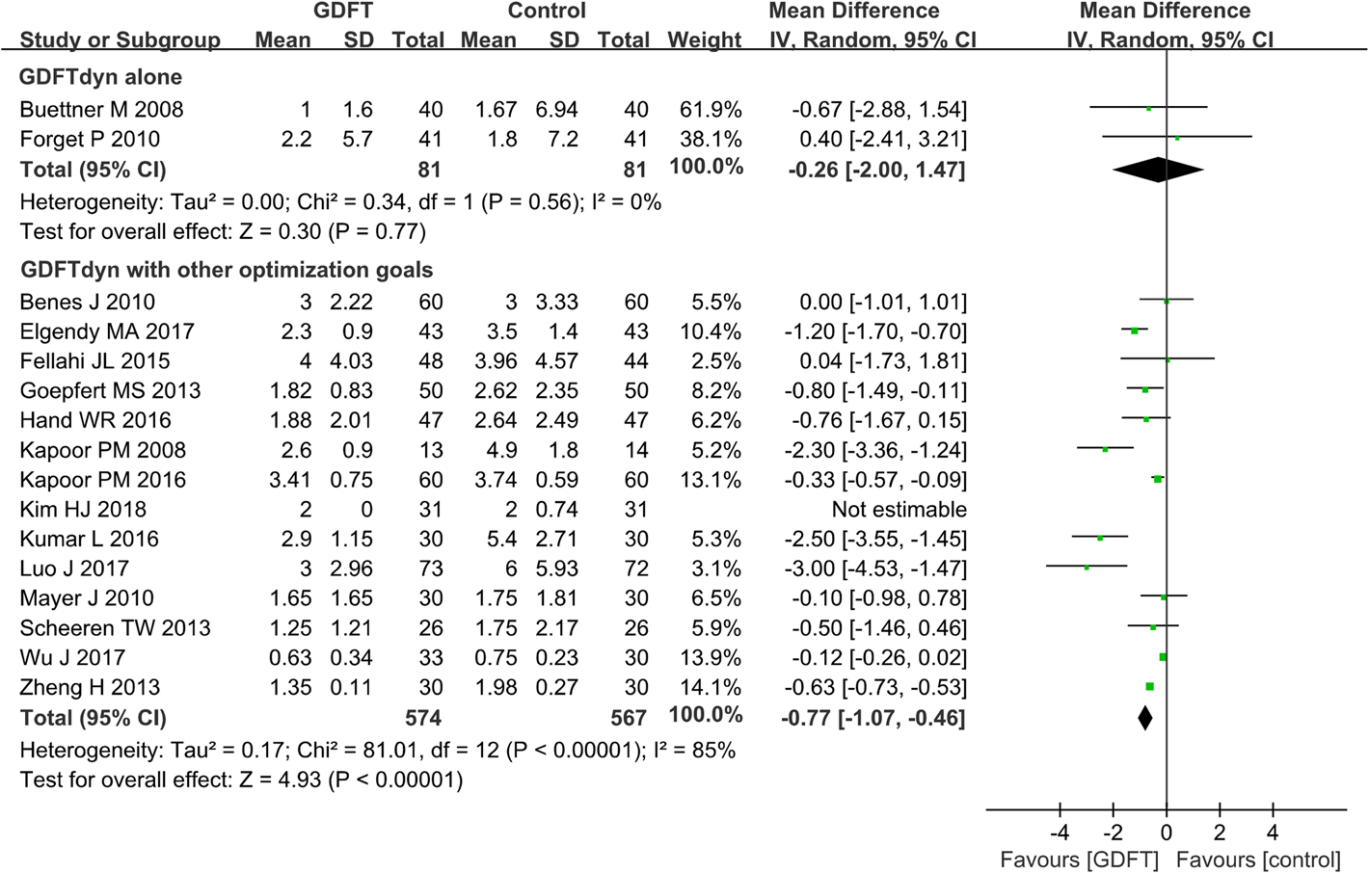
Length of stay in the ICU. GDFT, goal-directed fluid therapy

**Fig. 5:**
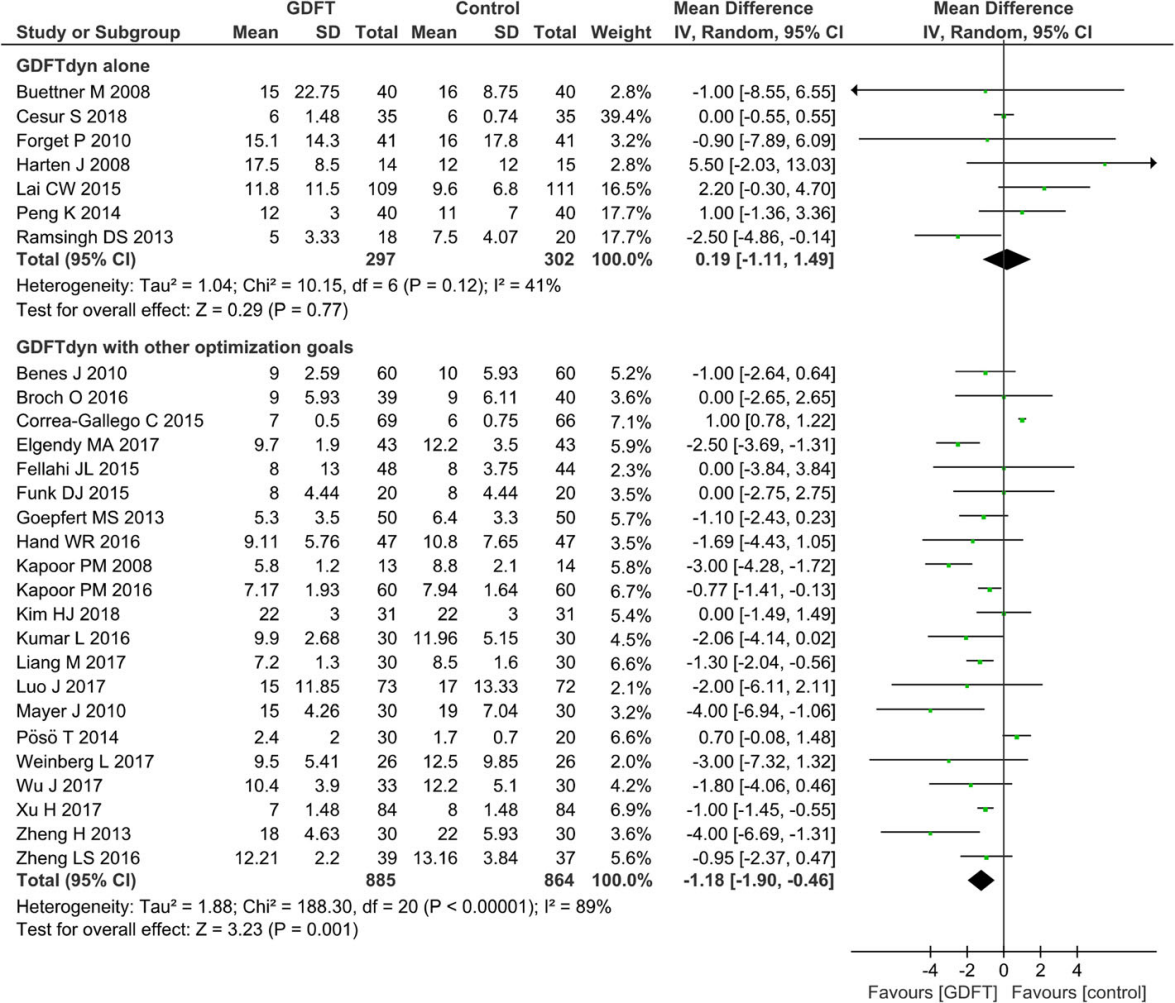
Length of stay in hospital. GDFT, goal-directed fluid therapy

Serum lactate concentration was significantly lower in patients managed with GDFTdyn alone (MD − 0.21 mmol/L, 95% CI (− 0.39, − 0.03), *P* = 0.02, I^2^ = 82%) (Fig. 3). However, no significant difference was found between two groups in any organ-specific morbidity (Table 2), length of stay in ICU (MD -0.26d, 95% CI (− 2.00, 1.47), *P* = 0.77, I^2^ = 0%) (Fig. 4) and hospital (MD 0.19d, 95% CI (− 1.11, 1.49), P = 0.77, I^2^ = 41%) (Fig. 5). The reduction in serum lactate concentration persisted in non-cardiac surgery, high-risk patients, fluid management without inotropes and minimally invasive monitoring device subgroups. No significant difference was found in length of stay in ICU and hospital among any subgroup analyses (Table 3).

**Table 2:** Meta-analysis of organ-specific morbidity between the GDFTdyn and standard fluid therapy group

Events	Studies, *n*	Patients (GDFT), *n*	Events (GDFT), *n*	Patients (control), *n*	Events (control), *n*	OR	95%CI	*P* value	References
Neurological events									
Stroke									
Analysis 2	7	292	3	286	10	0.38	(0.13, 1.13)	0.08	[13, 29, 34, 35, 43, 44, 49]
Cardiovascular events									
Arrhythmia									
Analysis 1	2	57	4	56	6	0.59	(0.16, 2.25)	0.44	[33, 36]
Analysis 2	14	513	37	504	57	0.58	(0.37, 0.92)	0.02*	[13, 21, 23, 24, 27, 29, 30, 32, 34, 35, 43, 44, 48, 49]
Myocardial infarction									
Analysis 2	10	423	8	416	23	0.35	(0.16, 0.76)	0.008*	[13, 20, 21, 23, 24, 30, 34, 35, 48, 49]
Heart failure/cardiovascular dysfunction									
Analysis 1	2	57	0	56	2	0.17	(0.01, 3.73)	0.26	[33, 36]
Analysis 2	9	403	7	400	25	0.31	(0.14, 0.67)	0.003*	[13, 29, 32, 34, 35, 43, 45, 48, 49]
Pulmonary events									
ALI/ARDS									
Analysis 1	2	57	2	56	5	0.4	(0.09, 1.86)	0.24	[33, 36]
Analysis 2	3	170	1	170	10	0.13	(0.02, 0.74)	0.02*	[13, 43, 45]
Pneumonia									
Analysis 1	2	57	6	56	8	0.69	(0.22, 2.15)	0.53	[33, 36]
Analysis 2	10	423	26	420	58	0.4	(0.24, 0.65)	0.0002*	[13, 23, 29, 30, 34, 35, 43, 45, 47, 49]
Pulmonary embolism									
Analysis 1	1	17	0	16	1	0.3	(0.01, 7.79)	0.47	[33]
Analysis 2	6	257	0	253	2	0.31	(0.03, 3.04)	0.31	[13, 29, 30, 34, 35, 44]
Abdominal events									
GIT bleeding									
Analysis 1	3	98	5	97	5	0.98	(0.27, 3.57)	0.98	[22, 33, 36]
Analysis 2	3	116	1	116	2	0.66	(0.11, 4.03)	0.65	[13, 35, 43]
GIT obstruction									
Analysis 1	1	17	0	16	1	0.3	(0.01, 7.79)	0.47	[33]
Analysis 2	5	170	4	170	5	0.83	(0.24, 2.79)	0.76	[13, 23, 30, 35, 48]
Renal events									
AKI									
Analysis 1	3	190	7	192	14	0.49	(0.19, 1.23)	0.13	[22, 31, 36]
Analysis 2	10	444	16	444	25	0.6	(0.31, 1.17)	0.14	[13, 17, 23, 24, 30, 34, 43–45, 47]
Renal failure with dialysis									
Analysis 1	2	81	2	81	0	3.08	(0.31, 30.19)	0.34	[22, 36]
Analysis 2	7	380	7	381	8	0.87	(0.32, 2.39)	0.79	[13, 17, 18, 20, 27, 34, 45]

Analysis 1: goal-directed fluid therapy based on dynamic parameters (GDFTdyn) alone versus standard fluid therapy; analysis 2: GDFTdyn with other optimization goals versus standard fluid therapy

*AKI* acute kidney injury, *ALI/ARDS* acute lung injury/acute respiratory distress syndrome, *CI* confidential interval, *GDFT* goal-directed fluid therapy, *GDFTdyn*, *GIT* gastrointestinal, *OR* odds ratio

*P < 0.05

**Table 3:** Subgroup analyses of clinical outcomes between the GDFTdyn and standard fluid therapy group

Subgroups	Analysis 1				Analysis 2			
	Studies, *n*	OR/MD	95%CI	*P* value	Studies, *n*	OR/MD	95% CI	*P* value
Short-term mortality								
Surgery								
Non-cardiac	6	0.85	(0.32, 2.24)	0.74	11	0.49	(0.24, 1.00)	0.05
Cardiac	–	–	–	–	2	0.35	(0.09, 1.36)	0.13
risk								
High	5	0.69	(0.25, 1.93)	0.48	12	0.45	(0.24, 0.85)	0.01*
Moderate	1	5.25	(0.24, 112.8)	0.29	–	–	–	–
Fluid/inotropes								
Fluid	6	0.85	(0.32, 2.24)	0.74	2	0.96	(0.04, 23.99)	0.98
Fluid+inotropes	–	–	–	–	11	0.42	(0.22, 0.82)	0.01*
Monitoring devices								
Minimally invasive	5	0.69	(0.25, 1.93)	0.48	13	0.45	(0.24, 0.85)	0.01*
Non-invasive	1	5.25	(0.24, 112.8)	0.29	–	–	–	–
Overall morbidity								
Surgery								
Non-cardiac	3	1.03	(0.31, 3.37)	0.97	14	0.4	(0.28, 0.59)	<0.00001*
Cardiac	–	–	–	–	1	0.4	(0.15, 1.06)	0.07
risk								
High	3	1.03	(0.31, 3.37)	0.97	14	0.4	(0.27, 0.58)	<0.00001*
Moderate	–	–	–	–	1	0.51	(0.18, 1.42)	0.2
Fluid/inotropes								
Fluid	3	1.03	(0.31, 3.37)	0.97	3	0.6	(0.30, 1.20)	0.15
Fluid+inotropes	–	–	–	–	12	0.37	(0.25, 0.55)	<0.00001*
Monitoring devices								
Minimally invasive	3	1.03	(0.31, 3.37)	0.97	14	0.4	(0.27, 0.58)	<0.00001*
Non-invasive	–	–	–	–	1	0.51	(0.17, 1.58)	0.24
Serum lactate concentration								
Surgery								
Non-cardiac	9	-0.21	(−0.39, −0.03)	0.02*	9	−0.67	(−1.14, −0.20)	0.005*
Cardiac	–	–	–	–	1	0.03	(−0.18, 0.24)	0.78
risk								
High	6	− 0.17	(− 0.32, − 0.02)	0.03*	10	− 0.6	(− 1.04, − 0.15)	0.009*
Moderate	3	− 0.19	(− 0.49, 0.11)	0.21	–	–	–	–
Fluid/inotropes								
Fluid	9	− 0.21	(− 0.39, − 0.03)	0.02*	1	− 0.4	(− 0.87, 0.07)	0.1
Fluid+inotropes	–	–	–	–	9	− 0.62	(− 1.10, − 0.13)	0.01*
Monitoring devices								
Minimally invasive	6	− 0.17	(− 0.32, − 0.02)	0.03*	9	− 0.68	(− 1.15, − 0.22)	0.004*
Non-invasive	3	− 0.19	(− 0.49, 0.11)	0.21	1	0.24	(− 0.22, 0.70)	0.31
length of stay in ICU								
Surgery								
Non-cardiac	2	−0.26	(−2.00, 1.47)	0.77	10	−0.77	(−1.15, − 0.39)	<0.0001*
Cardiac	–	–	–	–	4	−0.86	(− 1.68, − 0.04)	0.04*
Risk								
High	2	−0.26	(−2.00, 1.47)	0.77	12	−0.77	(−1.09, − 0.45)	< 0.00001*
Moderate	–	–	–	–	2	−0.76	(− 1.67, 0.15)	0.1
Fluid/inotropes								
Fluid	2	−0.26	(− 2.00, 1.47)	0.77	1	−0.5	(−1.46, 0.46)	0.3
Fluid+inotropes	–	–	–	–	13	−0.79	(−1.10, − 0.47)	<0.00001*
Monitoring devices								
Minimally invasive	1	−0.67	(−2.88, 1.54)	0.55	14	−0.77	(−1.07, − 0.46)	<0.00001*
Non-invasive	1	0.4	(−2.41, 3.21)	0.78	–	–	–	–
length of stay in hospital								
Surgery								
Non-cardiac	7	0.19	(−1.11, 1.49)	0.77	17	−1.13	(−1.94, −0.32)	0.006*
Cardiac	–	–	–	–	4	−1.42	(−2.63, − 0.21)	0.02*
Risk								
High	5	0.54	(−1.88, 2.96)	0.66	17	−1.45	(−2.37, −0.52)	0.002*
Moderate	2	−0.01	(−0.55, 0.54)	0.98	4	−0.33	(−1.47, 0.81)	0.58
Fluid/inotropes								
Fluid	7	0.19	(−1.11, 1.49)	0.77	2	0.16	(−1.74, 2.05)	0.87
Fluid+inotropes	–	–	–	–	19	−1.28	(− 1.82, −0.73)	<0.00001*
Monitoring devices								
Minimally invasive	5	0.54	(−1.88, 2.96)	0.66	20	−1.23	(−1.96, −0.49)	0.001*
Non-invasive	2	−0.01	(−0.55, 0.54)	0.98	1	0	(−2.65, 2.65)	1

Analysis 1: goal-directed fluid therapy based on dynamic parameters (GDFTdyn) alone versus standard fluid therapy; analysis 2: GDFTdyn with other optimization goals versus standard fluid therapy. Results for short-term mortality and overall morbidity are presented as odds ratio (OR) and 95% confidence interval (CI). Results on serum lactate concentration and length of stay in the ICU and in hospital are presented as mean difference (MD) and 95% CI

*ICU* intensive care unit

*P < 0.05

### Analysis 2: GDFTdyn with other optimization goals versus standard fluid therapy

#### Primary outcomes

Postoperative short-term mortality was reported in 13 studies including 1100 patients. Compared with standard fluid therapy, a significant reduction in short-term mortality was observed in favor of GDFTdyn with other optimization goals (OR 0.45, 95% CI (0.24, 0.85), *P* = 0.01, *I*^2^ = 0%) (Fig. 1). Sensitivity analysis excluding studies with high risk of bias also showed significant reduction in short-term mortality by GDFTdyn with other optimization goals (Additional file 4). Subgroup analyses showed that the reduction in short-term mortality was associated with high-risk patients, the use of fluid and inotropes, and minimally invasive monitoring devices (Table 3).

Postoperative overall morbidity was reported in 15 studies with 1330 patients. Overall morbidity was significantly reduced in patients managed with GDFTdyn and other optimization goals when compared with those managed with standard care (OR 0.41, 95% CI (0.28, 0.58), *P* < 0.00001, *I*^2^ = 48%) (Fig. 2). Sensitivity analysis excluding studies with high risk of bias also showed significant reduction in overall morbidity by GDFTdyn with other optimization goals (Additional file 5). Also, subgroup analysis showed that the reduction of overall morbidity was associated with non-cardiac surgery, high-risk patients, the use of fluid and inotropes, and minimally invasive monitoring devices (Table 3).

### Secondary outcomes

Compared with standard fluid therapy, serum lactate concentration (MD − 0.60 mmol/L, 95% CI (− 1.04, − 0.15), *P* = 0.009, *I*^2^ = 96%) (Fig. 3), incidence of cardiovascular complications (arrhythmia, OR 0.58, 95% CI (0.37, 0.92), *P* = 0.02, *I*^2^ = 0%; myocardial infarction, OR 0.35, 95% CI (0.16, 0.76), *P* = 0.008, *I*^2^ = 0%; heart failure/cardiovascular dysfunction, OR 0.31, 95% CI (0.14, 0.67), *P* = 0.003, *I*^2^ = 0%), pulmonary complications (ALI/ARDS, OR 0.13, 95% CI (0.02, 0.74), P = 0.02, *I*^2^ = 0%; pneumonia, OR 0.4, 95% CI (0.24, 0.65), *P* = 0.0002, *I*^2^ = 0%) (Table 2), and length of stay in the ICU (MD − 0.77d, 95% CI (− 1.07, − 0.46), *P* < 0.0001, *I*^2^ = 85%) (Fig. 4) and in hospital (MD − 1.18 days, 95% CI (− 1.90, − 0.46), *P* = 0.001, *I*^2^ = 89%) (Fig. 5) were significantly lower in patients managed with GDFTdyn with other optimization goals. The reduction in serum lactate concentration and length of stay in the ICU and in hospital persisted in high-risk patients, and in subgroups receiving fluid with inotropes and minimally invasive monitoring devices (Table 3).

## Discussion

The current study demonstrated that GDFTdyn alone was not associated with improved clinical outcomes except for the reduction in serum lactate concentration. However, further analysis of studies evaluating GDFTdyn with other optimization goals (mainly CO or CI) in their intervention arm revealed that the combination was associated with significant reduction in short-term mortality, overall morbidity, serum lactate concentration, cardiopulmonary complications, and length of stay in the ICU and in hospital.

Postoperative morbidity is as important as short-term mortality, for it might lead to loss of organ function and have an impact on long-term mortality [50]. Currently, evidence for the beneficial effects of GDFTdyn on mortality and morbidity has been inconsistent. Moreover, there is still no consensus on the most appropriate goals in GDFT strategies. Interestingly, our study revealed that optimization of fluid responsiveness by GDFTdyn alone was not associated with reduced mortality and morbidity. However, optimization of fluid responsiveness was found to be beneficial when it was in conjunction with other optimization goals (mainly CO or CI) to optimize tissue and organ perfusion. Increasing cardiac contractility produces an increase in the slope of the Frank-Starling curve, such that patients on the flat section of the original curve move to a steeper section of the new curve [51]. Therefore, by reaching the goals of GDFTdyn and CO/CI simultaneously, maximal stroke volume and adequate perfusion is achieved. Subgroup analyses also showed that the beneficial effects of GDFTdyn and other optimization goals persisted in patients using fluid and inotropes as the intervention. Another explanation for the improved clinical outcomes with the combination of GDFTdyn and CO/CI goals might be the gray zone of GDFTdyn endpoints. The gray zone of these dynamic variables has been considered unable to reliably predict fluid responsiveness [52, 53]. Although we could not identify the exact proportion of patients with a gray zone value in the studies included in our analysis, reaching CO/CI goals might prevent these patients from organ hypoperfusion. Our results contradicted a previous meta-analysis, which indicated a benefit of GDFTdyn compared to standard fluid therapy in reducing incidence of postoperative morbidity [5]. In their meta-analysis, 8 of 14 studies combined GDFTdyn endpoints with other optimization goals as interventions. Mixing studies on GDFTdyn alone with those on GDFTdyn with other optimization goals might lead to inaccurate or even erroneous conclusions.

High-risk patients undergoing surgery are thought to have higher oxygen demand and limited cardiopulmonary reserve. There is concern about GDFT-related cardiopulmonary complications in high-risk patients. Opposingly, we found that the improved clinical outcomes of GDFTdyn with CO/CI goals persisted in high-risk patients. Especially, in the analysis of organ-specific morbidity, cardiopulmonary complications were significantly reduced by the combined goals. Another meta-analysis on high-risk surgery also showed the use of fluid and inotropes reduced the incidence of cardiac arrhythmia without increasing the incidence of acute pulmonary edema [8]. It seems that maximizing stroke volume and oxygen delivery is beneficial especially for high-risk patients, which might be attributed to improved tissue perfusion and cardiac performance.

Serum lactate concentration could serve as a sensitive biochemical variable of oxygen debt. The association between decreased serum lactate and a reduction in postoperative complications was found in previous studies [54]. In the current study, significant reduction in serum lactate and postoperative morbidity were also observed in the group with GDFTdyn and other optimization goals. However, in the GDFTdyn-alone group, serum lactate was lowered but reduction in postoperative morbidity was not observed. The reduction in serum lactate by GDFTdyn alone (− 0.21 mmol/L) was much less than that by GDFTdyn with other optimization goals (− 0.60 mmol/L). It might imply that GDFTdyn alone was less effective in correcting tissue hypoperfusion without other optimization goals.

Length of stay in the ICU and in hospital were also shorter in patients managed with GDFTdyn with other optimization goals but not in those managed with GDFTdyn alone, which was similar to the results for postoperative morbidity. It is possible that the significant reduction in length of stay mostly is attributed to the lower incidence of postoperative complications. The heterogeneity of length of stay in the ICU and in hospital in the group with GDFTdyn and other optimization goals was greater than 75%. It might be attributed to the enormous change in the protocols and discharge criteria in the ICU and in hospital in recent years. Additionally, different units of measurement (days or hours) of length of stay in the ICU reported in different studies might also contribute to the heterogeneity.

Since the meta-analysis has several notable limitations, the results should be interpreted with caution. The main limitation was the clinical heterogeneity among different populations, surgical procedures, and monitoring devices. We tried to address the issue by the following measures. First, we divided the interventions into two groups and conducted two separate analyses (GDFTdyn alone versus standard fluid therapy and GDFTdyn with other optimization goals versus standard fluid therapy). Second, we conducted subgroup analyses according to the type of surgery (cardiac and non-cardiac), patient risk (high or moderate), fluid management (fluid with or without inotropes), and monitoring devices (minimally invasive or non-invasive). Finally, we used a random effect model to guarantee the robustness of the results and conclusions. Another limitation was failing to demonstrate a relationship between the year of publication of the included studies and the treatment effect. The included studies in the current meta-analysis spanned a long period of time. During this period, goal-directed fluid therapy has evolved rapidly and changed drastically. Also, fluid management in the postoperative period also has an important impact on clinical outcomes. However, postoperative fluid therapy regimes were not stated clearly in the included studies, making it difficult to evaluate the effects of them on perioperative outcomes.

## Conclusions

Based on the available data, we conclude that optimizing fluid responsiveness by GDFTdyn alone is not sufficient to improve clinical outcomes among patients undergoing surgery. However, the combination of GDFTdyn and other optimization goals to improve tissue and organ perfusion is associated with improved clinical outcomes. Patients managed with the combination of GDFTdyn and CO/CI goals might derive most benefit. High quality evidences with adequate statistical power and rigorous methodology are urgently needed to verify the beneficial effects of GDFT combined goals on clinical outcomes of patients undergoing surgery. Further researches are required to determine the most beneficial protocol and timing of GDFT strategies among different type of surgery (cardiac and non-cardiac) and different surgical populations (high or moderate risk).

## Additional files

Additional file 1: PRISMA checklists. (DOCX 18 kb)

Additional file 2: Flow chart of literature searching, reviewing and selection. (TIF 512 kb)

Additional file 3 Risk of bias summary presenting judgments for each risk of bias item for each included study. (TIF 898 kb)

Additional file 4: Forest plot for short-term mortality among studies with low or moderate risk of bias. (TIF 869 kb)

Additional file 5: Forest plot for overall morbidity among studies with low or moderate risk of bias. (TIF 809 kb)

## Abbreviations

AKI: Acute kidney injury
ALI/ARDS: Acute lung injury/acute respiratory distress syndrome
CI: Confidence interval
CIx: Cardiac index
CO: Cardiac output
CVP: Central venous pressure
DO_2_: Oxygen delivery
GDFT: Goal-directed fluid therapy
GDFTdyn: Goal-directed fluid therapy based on dynamic variables
GIT: Gastrointestinal
Hb: Hemoglobin
Hct: Red blood cell specific volume
ICU: Intensive care unit
MD: Mean difference
NR: Not referred
O_2_ER: O_2_ extraction rate
OR: Odds ratio
PaO_2_: Partial pressure of oxygen
PPV: Pulse pressure variation
PRISMA: Preferred Reporting Items for Systematic Reviews and Meta-Analyses
PVI: Pleth variability index
RCT: Randomized controlled trials
SAP: Systolic arterial pressure
ScVO_2_: Systemic central venous oxygen saturation
SPV: Systolic pressure variation
SVI: Stroke volume index
SVR: Systemic vascular resistance
SVRI: Systemic vascular resistance index
SVV: Stroke volume variation
T: Temperature
