# Supplementary figures and images for: Is goal-directed fluid therapy based on dynamic variables alone sufficient to improve clinical outcomes among patients undergoing surgery? A meta-analysis
Source: Crit Care. 2018 Nov 14;22:298. doi: 10.1186/s13054-018-2251-2 (PMC6237035; doi:10.1186/s13054-018-2251-2)

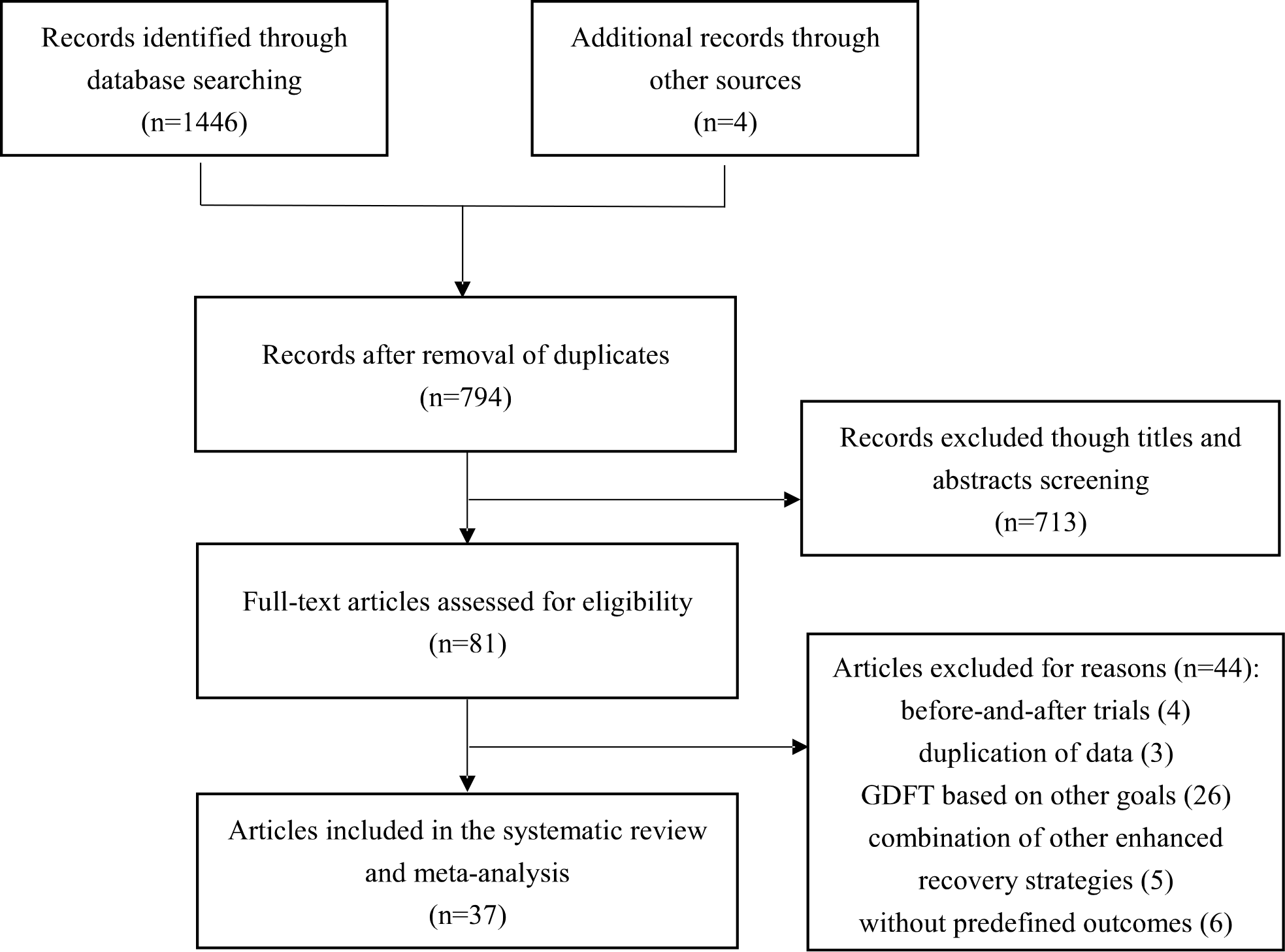

Supplement: Supplementary file 2 — Flow chart of literature searching, reviewing and selection. (TIF 512 kb) [file 13054_2018_2251_MOESM2_ESM.tif]

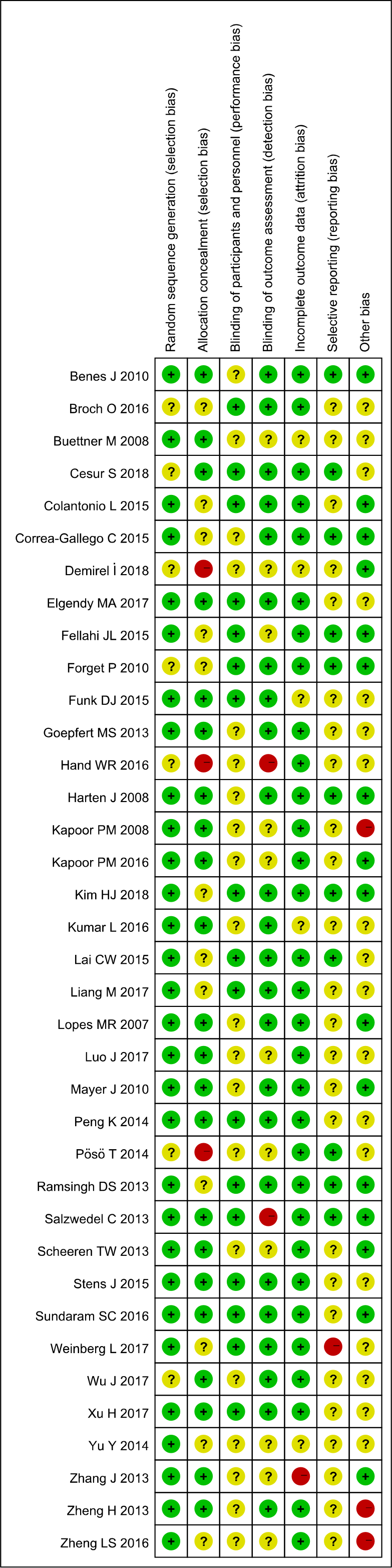

Supplement: Supplementary file 3 — Risk of bias summary presenting judgments for each risk of bias item for each included study. (TIF 898 kb) [file 13054_2018_2251_MOESM3_ESM.tif]

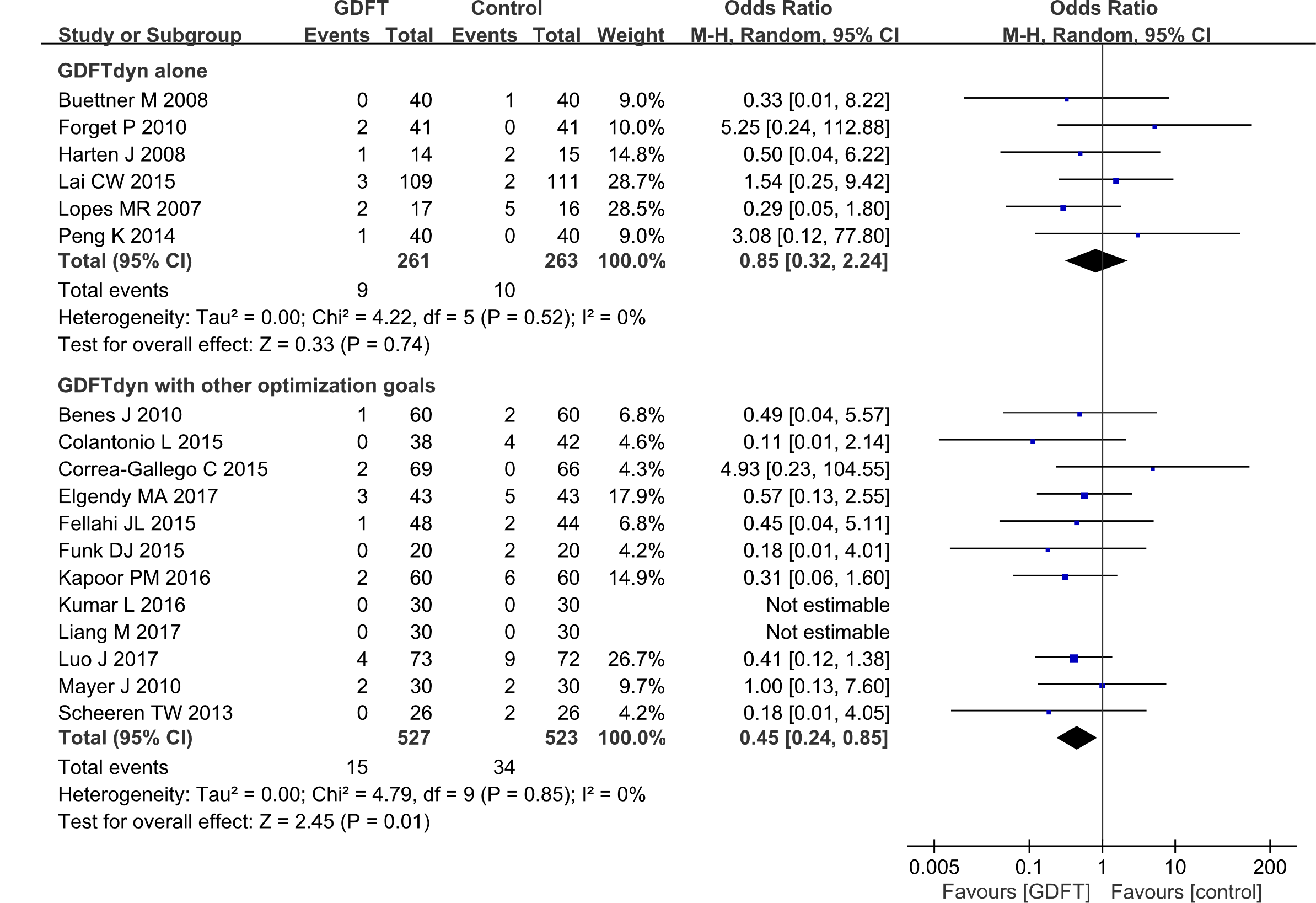

Supplement: Supplementary file 4 — Forest plot for short-term mortality among studies with low or moderate risk of bias. (TIF 869 kb) [file 13054_2018_2251_MOESM4_ESM.tif]

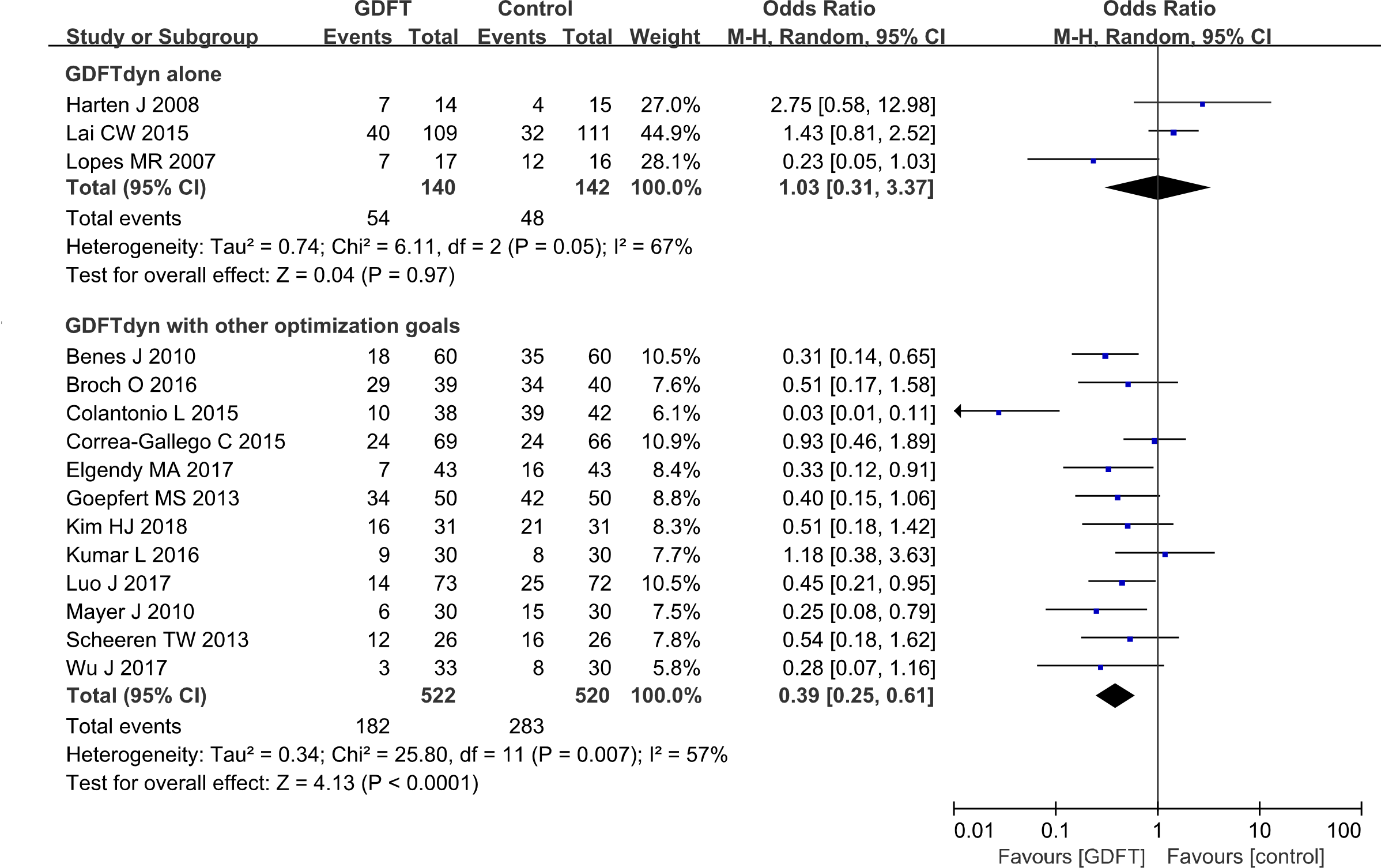

Supplement: Supplementary file 5 — Forest plot for overall morbidity among studies with low or moderate risk of bias. (TIF 809 kb) [file 13054_2018_2251_MOESM5_ESM.tif]
